# Quantitative and Sensitive Detection of Chloramphenicol by Surface-Enhanced Raman Scattering

**DOI:** 10.3390/s17122962

**Published:** 2017-12-20

**Authors:** Yufeng Ding, Xin Zhang, Hongjun Yin, Qingyun Meng, Yongmei Zhao, Luo Liu, Zhenglong Wu, Haijun Xu

**Affiliations:** 1 Beijing Key Laboratory of Bioprocess, Beijing University of Chemical Technology, Beijing 100029, China; 2015200888@mail.buct.edu.cn (Y.D.); yinhj@mail.buct.edu.cn (H.Y.); mengqy@mail.buct.edu.cn (Q.M.); liuluo@mail.buct.edu.cn (L.L.); 2 College of Science, Beijing University of Chemical Technology, Beijing 100029, China; 3 Engineering Research Center for Semiconductor Integrated Technology, Institute of Semiconductors, Chinese Academy of Sciences, Beijing 100083, China; ymzhao@semi.ac.cn; 4 Analytical and Testing Center, Beijing Normal University, Beijing 100875, China; wuzl@bnu.edu.cn

**Keywords:** surface-enhanced Raman scattering, Au nanoparticles, chloramphenicol, quantitative detection

## Abstract

We used surface-enhanced Raman scattering (SERS) for the quantitative and sensitive detection of chloramphenicol (CAP). Using 30 nm colloidal Au nanoparticles (NPs), a low detection limit for CAP of 10^−8^ M was obtained. The characteristic Raman peak of CAP centered at 1344 cm^−1^ was used for the rapid quantitative detection of CAP in three different types of CAP eye drops, and the accuracy of the measurement result was verified by high-performance liquid chromatography (HPLC). The experimental results reveal that the SERS technique based on colloidal Au NPs is accurate and sensitive, and can be used for the rapid detection of various antibiotics.

## 1. Introduction

Chloramphenicol (CAP), a common broad-spectrum antibiotic, is widely used in animal husbandry and aquaculture for its efficient inhibition of bacterial growth. Nonetheless, CAP brings serious adverse effects on human digestive and hematopoietic systems once its residues enter the body through the diet [1,2]. Moreover, CAP accumulates easily in the liver when it is absorbed in the body. Even after being discharged into the environment, it cannot degrade in the short-term [3,4], resulting in a lasting environmental pollution. For these reasons, CAP has been banned from animal feed and treatment in many countries, including China, the USA, Australia, and many European countries [5,6].

However, in reality CAP is still illegally used by many businesses pursuing economic interests, and what is worse, CAP residues are difficult to detect rapidly. Traditional methods for CAP detection such as high-performance liquid chromatography (HPLC), gas chromatography, and liquid chromatography mass spectrometry (LCMS) usually suffer from expensive, time-consuming, and complex analytical procedures [7,8,9,10]. Thus, developing methods to rapidly detect CAP is of great significance.

In recent years, the surface-enhanced Raman scattering (SERS) technique derived from the localized surface plasmon resonance of metallic nanostructures has received great interest owing to its nondestructive testing behavior and ultra-high chemical sensitivity [11,12], with applications such as structural analysis and biological sensing, and even drug safety and environmental analysis [13,14]. In clinical chemistry and some environmental fields, SERS is also a powerful spectroscopic tool [15,16]. Various SERS substrates have been designed by researchers to meet the need for substance detection, and they display a variety of testing performances; these include silver nanoparticles (NPs) [17], graphene/silver/laser-textured Si [18], and Au core-Ag shell NPs film [19]. However, colloidal Ag NPs tend to be easily oxidized, and the preparation processes of graphene/silver/laser-textured Si and Au core-Ag shell NPs film are complicated and tedious [17,18,19]. Colloidal Au NPs—only composed of the single precious metal—could be a promising SERS-active substrate with a simple preparation process, high sensitivity, repeatability, and biocompatibility, excellent stability and enhancement performance, and extremely strong and tunable surface plasmon resonance (SPR) from visible and near-infrared spectral regions [20,21]. In this study, colloidal Au NPs were used as SERS-active substrate to detect the CAP concentration in several types of CAP eye drops, and the accuracy of the testing results was verified by HPLC.

## 2. Experimental Section

### 2.1. Materials and Instruments

Chloroauric acid (HAuCl_4_), sodium citrate, and AgNO_3_ were purchased from Shanghai Aladdin Regent Co. Ltd. (Shanghai, China), rhodamine 6G (R6G) was purchased from J&K Scientific LTD. Pure CAP powder (99.7%) was purchased from Dr. Ehrenstorfer Gmbh (Augsburg, Germany). Three types of CAP eye drops (indicated as samples 1, 2, and 3) were produced by Shandong Boshilun Furuida Pharmaceutical Co. Ltd., Shandong, China, Handan Kangye Pharmaceutical Co. Ltd. (Handan, China), and Guangdong Hongying Science and Technology Co. Ltd. (Guangzhou, China), respectively. Methyl alcohol, glacial acetic acid, and deionized water were from Beijing Chemical Works. All chemicals were of analytical grade and were used as received.

The typical morphologies and microstructures of Au NPs were investigated by transmission electron microscopy (TEM) (HT7700, HITACHI). The ultraviolet-visible (UV-Vis) absorption spectra were obtained by using a Shimadzu UV-3600 UV-Vis spectrophotometer. All Raman signals were obtained on the LabRAM ARAMIS Raman testing system. The HPLC spectrum was monitored by a Shimadzu LC-6A system.

### 2.2. Preparation of Colloidal Au NPs and CAP Solution

Colloidal Au NPs with average sizes of 10, 20, 30, 40, and 50 nm were prepared, respectively. The 10 nm colloidal Au NPs were prepared in the following method: 1.5 mL 1% *w*/*v* sodium citrate and 42.5 μL 0.1% *w*/*v* AgNO_3_ were added into 1 mL 20 mM HAuCl_4_ aqueous solution successively under vigorous stirring, and after stirring for 5 min the mixed solution was added into 47.5 mL boiling deionized water. After that, the mixed solution was heated to boiling and kept for 1 h under vigorous stirring to finish the preparation of colloidal Au NPs before it slowly cooled down to room temperature. The 20 nm colloidal Au NPs were prepared in the same procedures as above, but with the use of 1 mL 20 mM HAuCl_4_ aqueous solution, 0.7 mL 1% *w*/*v* sodium citrate, 42.5 μL 0.1% *w*/*v* AgNO_3_ solution, and 0.8 mL deionized water.

The 30, 40, and 50 nm colloidal Au NPs prepared by the same method as above aggregate excessively, so another protocol without using AgNO_3_ was tried to prepare them. To be more specific, the 30, 40, and 50 nm colloidal Au NPs were prepared by the following procedure: 1% *w*/*v* sodium citrate with a volume of 0.5, 0.4, and 0.35 mL, respectively, was added into 50 mL 0.25 mM boiling HAuCl_4_ aqueous solution under vigorous stirring. After that, the mixed solution was heated to boiling and kept for 18 min under vigorous stirring to finish the preparation of colloidal Au NPs before it slowly cooled down to room temperature.

The 0.0032 g pristine CAP powder was dissolved in 10 mL of deionized water as stock solution (10^−3^ M). After being ultrasonically vibrated for 10 min, the stock solution was gradually diluted in series with deionized water to obtain CAP solutions with the desired concentrations: 10^−4^, 10^−5^, 10^−6^, 10^−7^, and 10^−8^ M. The Raman spectrum of pure CAP was obtained by directly measuring the as-received CAP powder.

### 2.3. SERS Measurements

The Raman signals were obtained at room temperature on the LabRAM ARAMIS Raman system with 785 nm laser as excitation. The incident power was 0.325 mW, and the diameter of the light spot area was ~1 μm. Raman spectra were recorded with an accumulation time of 15 s, and the spectral resolution was 1 cm^−1^. The above parameters were the same for all Raman spectra in the case of no special instructions. All the data were averaged over 20 randomly selected positions.

## 3. Results and Discussion

### 3.1. Characterization of Colloidal Au NPs

**Figure 1:**
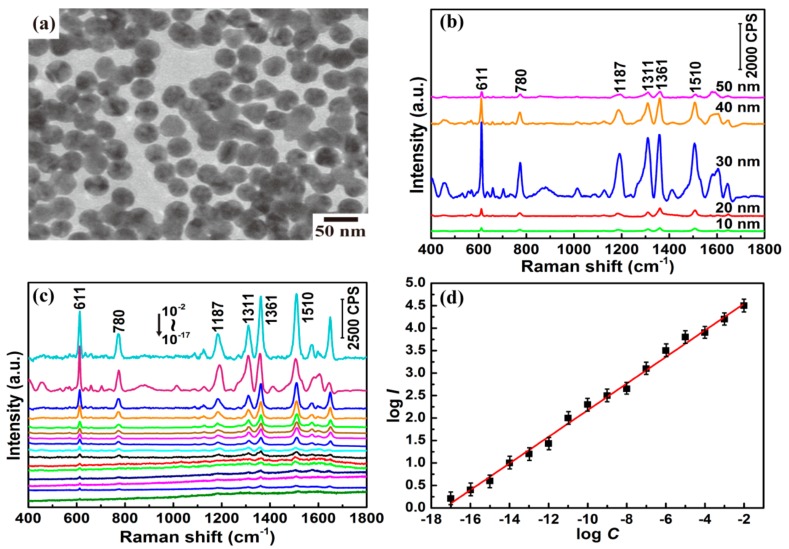
(**a**) Transmission electron microscopy (TEM) image of 30 nm colloidal Au nanoparticles (NPs); (**b**) surface-enhanced Raman scattering (SERS) spectra of 10^−3^ M rhodamine 6G (R6G) using colloidal Au NPs with different sizes (10, 20, 30, 40, and 50 nm); (**c**) SERS spectra of R6G with concentrations from 10^−17^ to 10^−2^ M (bottom to top, concentration successively increasing by a factor of 10) using 30 nm colloidal Au NPs. The intensity is multiplied five times for 10^−11^ to 10^−7^ M R6G and 10 times for 10^−17^ to 10^−12^ M R6G; (**d**) Linear relationship between log *I* of the band peak at 1361 cm^−1^ and log *C* based on the SERS data of R6G in (**c**).

To obtain excellent SERS enhancement, 10, 20, 30, 40, and 50 nm colloidal Au NPs were prepared. TEM was used to characterize the structures of the colloidal Au NPs. Figure S1 (in the Supplementary Materials) and Figure 1a show the TEM images of colloidal Au NPs with different sizes. From Figure S1a, it can be seen that the colloidal Au NPs were highly dispersed and had an average size of ~10 nm. This type of colloid will not have significant Raman enhancement because the density of the “hot spot” is too low. As shown in Figure S1c, the colloidal Au NPs aggregated and became larger (~50 nm), which resulted in a similarly weak Raman signal. Therefore, 30 nm colloidal Au NPs were chosen as the substrate to enhance the SERS signal, owing to the appropriate dispersibility and high density of the “hot spot”. The formation of colloidal Au NPs with different sizes from 10 to 50 nm can be reflected in Figure S1d, in which the UV-Vis absorption peak of the colloidal Au NPs shifts from 516 to 538 nm.

### 3.2. SERS Activities of Colloidal Au NPs

R6G is extensively used in SERS technique owing to its well-established vibrational features, and was used as the probe molecule to characterize the SERS performance of the colloidal Au NP substrate [22]. A 532 nm laser was first considered as excitation source for the SERS test, as this wavelength is closer to the electronic resonance wavelength of R6G [23]. Moreover, from the UV-Vis curves, the wavelength giving the highest surface plasmon bands is within 500–600 nm. However, as shown in Figure S2, for the detection of CAP, 785 nm excitation generates stronger Raman signal than the 532 and 633 nm excitation do, so 785 nm was finally chosen as the excitation for R6G detection to keep the consistency of experimental conditions. For the detection of R6G, solutions with different concentrations were added into the colloidal Au NPs with a 1:1 ratio in volume to obtain the mixtures. Figure 1b shows the Raman spectra of the mixtures of 10^−3^ M R6G and differently sized colloidal Au NPs. The Raman bands centered at about 611, 780, and 1187 cm^−1^ can be assigned to C-C-C ring in-plane bending, C-H out-of-plane bending, and C-H in-plane bending, respectively. The other features at about 1311, 1361, and 1510 cm^−1^ all originate from aromatic C-C stretching vibrations [24]. The mixture of R6G and 30 nm colloidal Au NPs showed the best SERS enhancement, so 30 nm colloidal Au NPs were chosen as the substrate to enhance the Raman signal for detecting R6G. To further examine the SERS activity of the substrate, the SERS spectra of the mixtures of R6G with a gradient concentration (10^−2^ to 10^−17^ M) and 30 nm colloidal Au NPs were recorded, and they are shown in Figure 1c. The Raman peaks of R6G are still quite clear, even for a concentration as low as 10^−17^ M. Because the Raman peak at ~1361 cm^−1^ is more intense than the other peaks, it was chosen to establish the quantitative relationship between the Raman intensity and the concentration of the probe molecule. Figure 1d shows the quantitative relationship curve between the integrated SERS intensity of the 1361 cm^−1^ peak (*I*) and the concentration of the R6G solution (*C*). When logarithm-scale axes are used, a linear response between log *I* and log *C* is obtained, which indicated that this linear relationship allowed for the determination of unknown concentration of R6G in solutions [25].

The time stability of the colloidal Au NPs was also investigated, and it was concluded that the lifetime of the SERS tags is dependent on the storage conditions. The colloidal Au NPs can be stored for several weeks with excellent stability and activity at low temperatures (e.g., 4 °C). The detailed information can be found in Figure S3 in the Supplementary Materials.

### 3.3. Quantitative Detection of CAP by SERS

**Figure 2:**
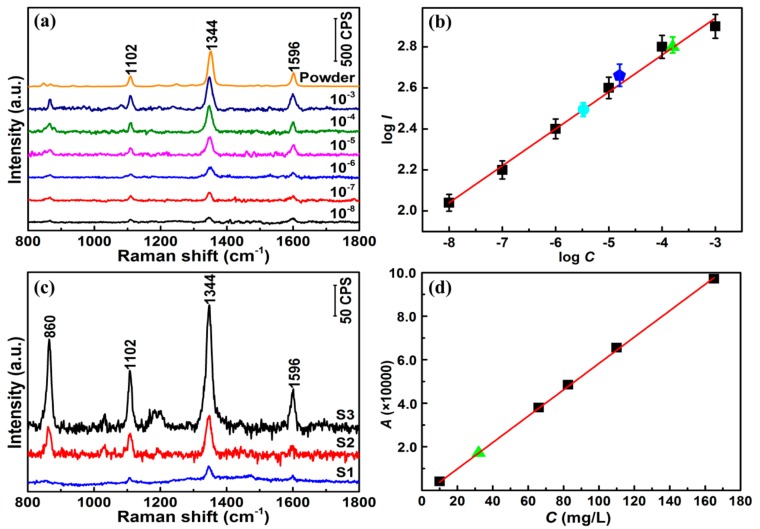
(**a**) SERS spectra of chloramphenicol (CAP) solid powder and CAP solutions with concentrations from 10^−8^ to 10^−3^ M using 30 nm colloidal Au NPs; (**b**) Linear relationship between log *I* of the band peak at 1344 cm^−1^ and log *C* based on the SERS data of the CAP solutions in (**a**) (six black spots). The cyan circle, blue pentagon, and green triangle represent the results for three different types of CAP eye drops below (samples 1, 2, and 3); (**c**) SERS spectra of three types of CAP eye drops using 30 nm colloidal Au NPs; (**d**) High-performance liquid chromatography (HPLC) linear relationship between *A* and *C* based on the data of pure CAP solutions with different concentrations (five black spots). The green triangle corresponds to sample 3.

CAP solutions with different concentrations and three types of CAP eye drops were added into colloidal Au NPs with a 1:1 ratio in volume to fabricate the mixtures, respectively. Figure S4 shows the Raman spectra of the mixtures of 10^−3^ M CAP and differently sized colloidal Au NPs. Owing to the excellent SERS enhancement, 30 nm colloidal Au NPs were chosen as the SERS substrate for the detection of CAP. Figure 2a shows the Raman spectra of the mixtures of CAP solutions ranging from 10^−3^ to 10^−8^ M and 30 nm colloidal Au NPs. Here, the Raman spectrum of the CAP solid powder is also detected and shown in Figure 2a. The Raman peaks of the CAP solutions coincide with the peaks of the CAP solid powder. The CAP molecule is composed of a nitro phenyl, a propylene glycol, and a dichloro ethyl amide group, as shown in Figure S5. The different structural components produce different Raman peaks. The Raman band at ~1102 cm^−1^ is assigned to the bending vibration of 14C-18O and the stretching vibration of C-H bond on the benzene ring. The Raman band at ~1344 cm^−1^ is assigned to the bending vibration of the -NO_2_ group, the C-H bond on the benzene ring, and the stretching vibration of the carbon chain. The Raman band at ~1596 cm^−1^ can be assigned to the stretching vibrations of 24C-25O and 24C-22N.

From Figure 2a, it can be seen that the Raman peaks of the CAP solutions are still distinct at a low concentration of 10^−8^ M. In addition, the Raman peak at ~1344 cm^−1^ shows the best Raman signal. Figure 2b shows the quantitative relationship curve between the integrated SERS intensity of the ~1344 cm^−1^ peak (*I*) and the concentration of the CAP solution (*C*). In Figure 2b, the six black squares are the data from Figure 2a. Each data point is the average of 20 independent experiments. When logarithm-scale axes are used, the response between log *I* and log *C* is linear, and linear regression gives the relationship log I=3.45+0.175×log C.

To confirm the accuracy of the linear relationship, CAP eye drop solutions with two random concentrations were prepared, 1 × 10^−5.50^ M (sample 1) and 1 × 10^−5.00^ M (sample 2), and SERS measurements were then performed. The Raman spectra are shown in Figure 2c. The log *I* values of these two samples were calculated, and they are shown in Figure 2b as a cyan circle (sample 1) and a blue pentagon (sample 2). Substituting the log *I* values into the formula logI=3.45+0.175×logC gives CAP concentrations of 1 × 10^−5.44^ M and 1 × 10^−4.80^ M for samples 1 and 2, respectively, which are close to the actual concentrations. This coincidence is shown well in Figure 2b, in which the cyan circle and blue pentagon are basically on the red fitted line. Therefore, the linear relationship can be used to determine the unknown concentration of CAP in solutions using this substrate.

To verify the accuracy of the method, another actual eye drop sample (sample 3) with an unknown concentration of CAP was analyzed by SERS and HPLC. The detected Raman spectrum of sample 3 is shown in Figure 2c, and the concentration was calculated to be 1 × 10^−3.80^ M using the formula logI=3.45+0.175×logC. This result is displayed in Figure 2b as a green triangle. On the other hand, according to the HPLC results of CAP solutions with five different concentrations shown in Figure 2d, the linear relationship A=−2255.65+605.56×C was obtained, where *A* and *C* represent the peak area and the concentration of CAP, respectively. The green triangle in Figure 2d is the result of sample 3. Based on this HPLC result, the concentration of sample 3 was determined to be 32.3 mg/L. This calculated value is in agreement with the calculated concentration (1 × 10^−3.80^ M) using the SERS linear regression formula, demonstrating the accuracy of the SERS method for detecting CAP.

## 4. Conclusions

In summary, homogenous and monodispersed colloidal Au NPs have been prepared and used as a low-cost SERS substrate for quantitative and rapid detection of CAP. The experimental results reveal that the 30 nm colloidal Au NP substrate exhibits excellent SERS performance and a low detection limit of 10^−8^ M for CAP. The characteristic Raman peak of CAP at 1344 cm^−1^ was used for rapid quantitative detection of CAP in three different types of CAP eye drops. HPLC spectrum verified the accuracy of the SERS method for detecting CAP. This method can be used for the quantitative and sensitive detection of CAP, and could possibly be applied to detect other molecular organic species.

## Supplementary Materials

The following are available online at www.mdpi.com/1424-8220/17/12/2962/s1. Figure S1: TEM images of 10 nm (a), 30 nm (b) and 50 nm (c) colloidal Au NPs, respectively; (d) The UV-Vis spectra of colloidal Au NPs with different sizes (10, 20, 30, 40 and 50 nm), Figure S2: SERS spectra of pristine CAP powder with 532, 633 and 785 nm laser as excitation, respectively, Figure S3: SERS spectra of the mixtures of 10^−2^ M R6G solution and 30 nm colloidal Au NPs: (a) The Au NPs are stored for 0 to 24 h at room temperature; (b) The Au NPs are stored for 1 to 45 days at 4 °C, Figure S4: SERS spectra of 10^−3^ M CAP solution using colloidal Au NPs with different sizes (10, 20, 30, 40 and 50 nm), Figure S5: Schematic diagram of the CAP molecule.
