# Supplementary material for: Quantitative and Sensitive Detection of Chloramphenicol by Surface-Enhanced Raman Scattering
Source: Sensors (Basel). 2017 Dec 20;17(12):2962. doi: 10.3390/s17122962 (PMC5751667; doi:10.3390/s17122962)
Supplement: Supplementary file 1 [file sensors-17-02962-s001.pdf]

Supplemental Material

Quantitative and sensitive detection of chloramphenicol by surface-enhanced Raman scattering

Yufeng Ding[1,2], Xin Zhang[1,2,*], Hongjun Yin[1,2], Qingyun Meng[1,2], Yongmei Zhao[3], Luo Liu[1], Zhenglong Wu[4] and Haijun Xu[1,2,*]

[1]Beijing Key Laboratory of Bioprocess, Beijing University of Chemical Technology, Beijing 100029, China; 2015200888@stud.buct.edu.cn (Y.D.); yinhj@mail.buct.edu.cn (H.Y.); mengqy@mail.buct.edu.cn (Q.M.); liuluo@mail.buct.edu.cn (L.L.)
[2]College of Science, Beijing University of Chemical Technology, Beijing 100029, China
[3]Engineering Research Center for Semiconductor Integrated Technology, Institute of Semiconductors, Chinese Academy of Sciences, Beijing 100083, China; ymzhao@semi.ac.cn
[4]Analytical and Testing Center, Beijing Normal University, Beijing 100875, China; wuzl@bnu.edu.cn
* Correspondence: zhxin@mail.buct.edu.cn (X.Z.); hjxu@mail.buct.edu.cn (H.X.)
Tel.: +86-10-6444-2357 (H.X.); Fax: +86-10-6443-5170 (H.X.)

Morphologies, microstructures and UV-Vis spectra of colloidal Au NPs

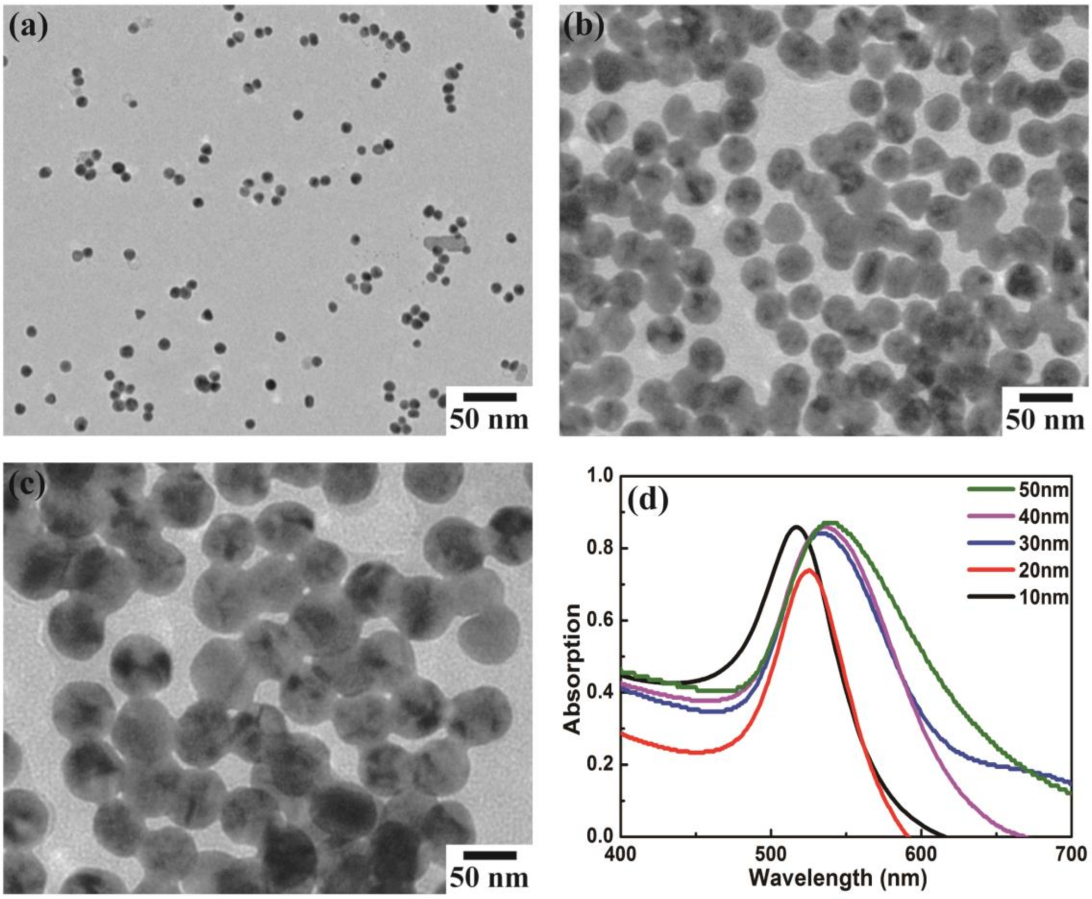

Figure S1. TEM images of 10 nm (**a**), 30 nm (**b**) and 50 nm (**c**) colloidal Au NPs, respectively; (**d**) The UV-Vis spectra of colloidal Au NPs with different sizes (10, 20, 30, 40 and 50 nm).

SERS activities of pristine CAP powder with 532, 633 and 785 nm laser as excitation

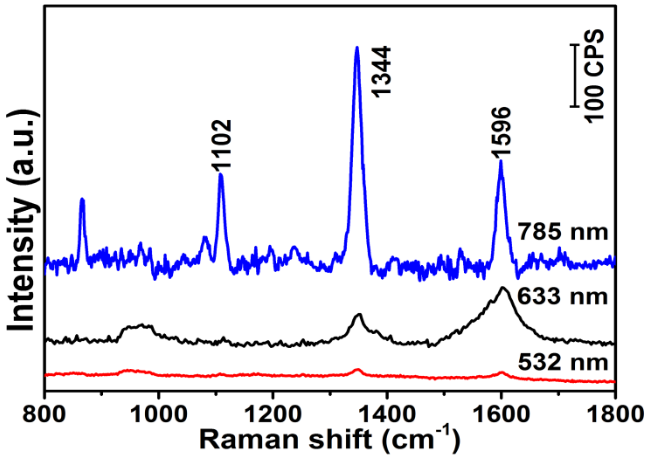

Figure S2. SERS spectra of pristine CAP powder with 532, 633 and 785 nm laser as excitation, respectively.

Lifetime of 30 nm colloidal Au NPs

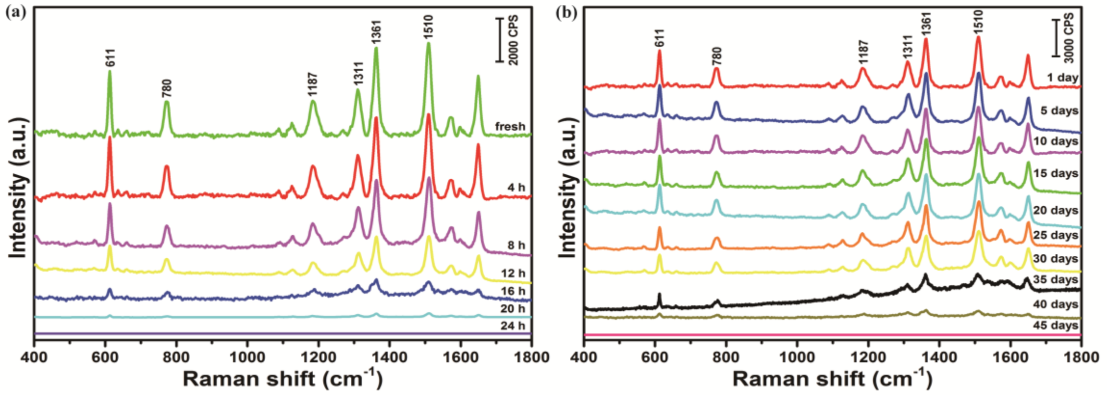

Figure S3. SERS spectra of the mixtures of 10^{-2} M R6G solution and 30 nm colloidal Au NPs: (**a**) The Au NPs are stored for 0 to 24 hours at room temperature; (**b**) The Au NPs are stored for 1 to 45 days at 4 °C.

SERS activities of CAP solution using colloidal Au NPs with different sizes

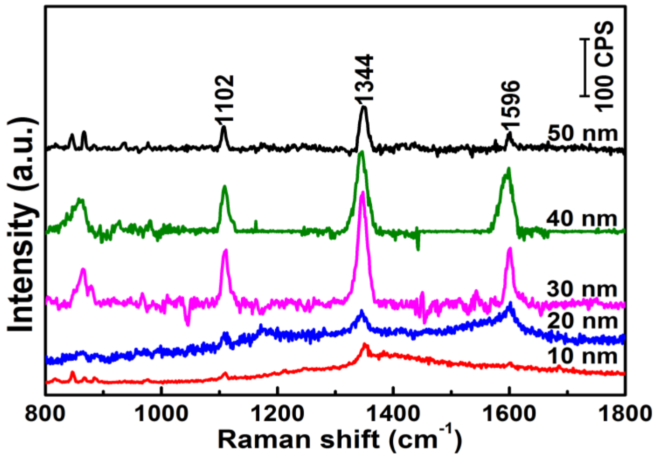

Figure S4. SERS spectra of 10^{-3} M CAP solution using colloidal Au NPs with different sizes (10, 20, 30, 40 and 50 nm).

Molecular structure of CAP

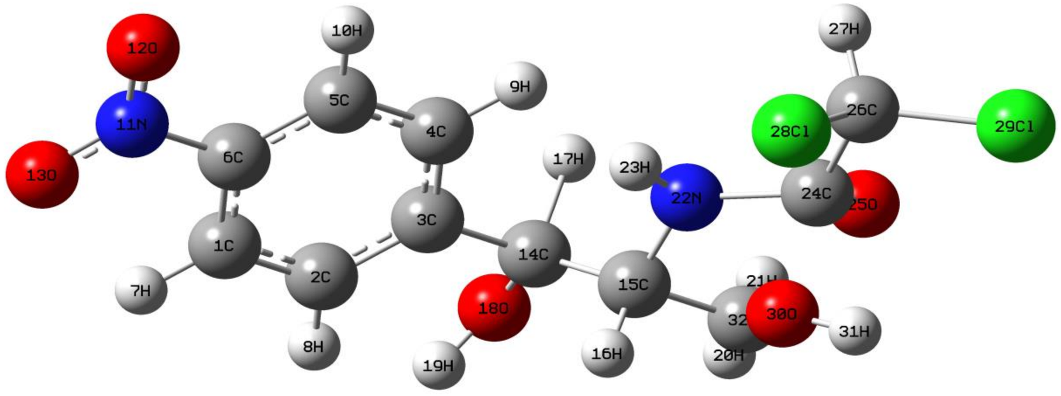

Figure S5. Schematic diagram of the CAP molecule.
